# Carbon emissions and low-carbon innovation in firms

**DOI:** 10.1371/journal.pone.0312759

**Published:** 2024-10-24

**Authors:** Jiao Ma

**Affiliations:** School of Accounting, Southwestern University of Finance and Economics, Chengdu, Sichuan Province, China; University of Science and Technology of China, CHINA

## Abstract

Most of the previous studies of environmental innovation focus on the impact of environmental innovation on carbon emissions. This study rarely examines the internal causes and mechanisms of influence of low-carbon innovation. This study focuses on the effect of carbon emissions on low-carbon innovation in firms. Using a panel data set of Chinese A-share firms, this study finds that the increase in carbon emissions promotes low-carbon innovation. This promoting effect comes from high carbon emissions increasing the pressure to reduce carbon emissions in firms and prompting firms to increase R&D investment, and the effect is more pronounced in firms with lower equity concentration or high-tech firms. It is also found that indirect carbon emissions do not promote low-carbon innovation, while other types of carbon emissions do. This study expands the research on the internal causes of low-carbon innovation in firms, examines the logic influencing low-carbon innovation in firms from the perspective of emission reduction motives and methods, reveals that global warming contains opportunities for the development of low-carbon innovation in firms, and provides a reference for optimizing the carbon emissions calculation system.

## Introduction

Climate change is one of the most serious global issues in recent years. In December 2015, the United Nations Climate Change Conference (Climate Conference in Paris) adopted the Paris Agreement, in which 195 parties agreed to reduce greenhouse gas emissions and keep the global average temperature rise “below 2°C above pre-industrial levels”. Against the backdrop of global warming, the green, low-carbon economy characterized by low energy consumption, low greenhouse gas emissions, and low environmental pollution has become a global hot topic [1], with its core being the low-carbon innovation aimed at breaking away from carbon-intensive economies [2]. As the world’s largest emitter of greenhouse gases, China is actively responding to global climate change. In the face of climate change, China has vigorously developed a green, low-carbon economy, accelerating the development and application of low-carbon innovation. On one hand, China’s energy-related emissions have grown rapidly over the past few decades, with carbo. On the other hand, the number of green, low-carbon patent authorizations in China continues to grow with an average annual increase of 6.5%. From 2016 to 2021, the State Intellectual Property Office (SIPO) authorized 160,000 green, low-carbon patents, accounting for 34.0% of the global total (SIPO, 2022). Does the increasing carbon emissions stimulate the growth of China’s low-carbon innovation? Considering that firms are the main drivers of low-carbon innovation, do firms’ carbon emissions promote the emergence of low-carbon innovation?

Existing literature mostly explores the factors influencing low-carbon innovation in firms from an external perspective. Stakeholders such as governments, suppliers, finance, and intermediary institutions significantly affect low-carbon innovation in firms [3, 4]. Stakeholders with carbon concerns exert pressure on firms to promote low-carbon innovation [5]. Carbon systems, tax competition, and rewards for low-carbon innovation can also affect low-carbon innovation [6, 7]. A small number of studies use surveys and literature reviews to discuss the role of internal factors in low-carbon innovation from the perspectives of organizational structure and financiers [8, 9]. Current research lacks an exploration of the relationship between internal factors within firms and low-carbon innovation based on large-sample data. Will the behavior of carbon emissions, which represents the firms’ fulfillment of carbon responsibility, force firms to engage in low-carbon innovation? What is the path of its influence?

This study attempts to examine the impact and mechanism of carbon emissions on low-carbon innovation in firms based on a large sample of data, to understand the role of firms in addressing climate change. The study finds that carbon emissions promote low-carbon innovation in firms. Mechanism analysis reveals that carbon emissions reduction pressure and R&D investment are the intermediary factors through which carbon emissions affect low-carbon innovation. Further analysis indicates that the promoting effect is more pronounced in firms with low equity concentration and high-tech firms. The indirect carbon emissions from the purchase of electricity, heat, and steam do not promote low-carbon innovation, while other types of carbon emissions do have a promoting effect on low-carbon innovation.

Existing research on innovation in firms often uses green patents as a proxy for low-carbon innovation [10, 11]. The scope of green patents is broad and not limited to low-carbon aspects. This study focuses more specifically on low-carbon patents. The innovative aspects of this study are in the following aspects. First, it extends the literature on the economic consequences of carbon emissions in firms and rarely analyzes the internal causes of low-carbon innovation in firms based on large-sample data. Most previous studies focus on the impact of carbon emissions on environmental penalties, stock prices, capital costs, and public image of firms [12–17], and focus on analyzing the negative consequences of carbon emissions in firms from the perspective of the external environment. This study, for the first time, examines the economic consequences of carbon emissions in firms from the perspective of internal motivation, specifically low-carbon innovation, and examines the positive actions of firms in response to climate change. The results of this study show that the process before reaching a carbon peak will also be accompanied by an increase in low-carbon innovation in firms, and we should calmly accept the process of carbon growth and actively respond.

Second, previous research on low-carbon innovation has rarely delved deeply into the mechanisms of impact. This study, for the first time, identifies carbon emissions reduction pressure and R&D investment as intermediary factors influencing low-carbon innovation in firms from the perspective of emission reduction motives and methods. It suggests that firms make efficient decisions after weighing the costs and benefits.

Third, the research direction of this study differs from the main research directions of the impact of green technologies on carbon emissions [18]. This study is related to the research of Su and Moaniba (2017) [19], Wang et al. (2020) [20], and Pan et al. (2021) [21], but it differs. First, unlike previous studies that focus on the national, provincial, and city levels of data, this study focuses on the firm level, specifically the carbon emissions and low-carbon innovation of listed firms. Because firms are an important subject of low-carbon innovation, research on firm-level data is more conducive to forming replicable corporate governance experience.

Fourth, while previous studies explore the impact of heterogeneous innovation [22, 23], they do not discuss the heterogeneity of carbon emissions. This study investigates the heterogeneity in the effects of different types of carbon emissions on low-carbon innovation in firms, which has some policy implications.

Last, carbon emissions are not only a problem for China but a global issue. The conclusions drawn from this study, which takes China as the research setting, help to promote the experience of the world’s largest carbon-emitting country in dealing with climate change to other countries, providing empirical evidence for better climate change response.

## Literature review

From the perspective of a firm’s production and business operations, carbon emissions include three scopes: Scope 1 is the direct carbon emissions from the production process; scope 2 is the indirect carbon emissions from purchased electricity, heat, and steam; scope 3 is the other indirect carbon emissions from the firm’s value chain upstream and downstream. In recent years, research on the economic consequences of carbon emissions has sparked heated discussions in academia. Existing studies find that there is a negative correlation between the degree of corporate pollution and the bank loans obtained [24]. Firms with lower carbon emissions can obtain more investment and stockholding intentions, and investors find it difficult to hold the bonds of firms with high carbon emissions for a long time [25]. Carbon emissions increase the downside risk of put option prices, and public concern about climate change increases the cost of guarding against downside tail risks [25]. Stocks of carbon-intensive assets and firms with high carbon emissions obtain higher returns because investors require a higher risk premium for them [15, 26], and highly polluting firms are more likely to face environmental penalties, resulting in a pollution premium [27]. Firms in the US and the UK with high carbon emissions tend to have weaker performance [16, 28]. Greenhouse gas emissions are an important factor for investors to consider when valuing firms, and higher carbon emissions intensity is associated with negative stock price reactions and news [14, 15]. High carbon risk is associated with high credit risk and low investment efficiency [17, 29]. It can be seen that most studies find that high carbon emissions will have a negative impact on the development of firms, and ignore the driving force it may generate for the development of firms. Additionally, the existing literature does not distinguish the economic consequences of different types of carbon emissions in firms.

Low-carbon innovation can reduce carbon emissions from the combustion of fossil fuels, develop clean energy sources such as wind and solar energy, transform production equipment with high carbon emissions, and capture and store emitted carbon dioxide [21]. Compared to general innovation, low-carbon innovation has its unique patterns. Studies based on patent data indicate that low-carbon innovation has higher uncertainty than general technological innovation [30]. Interviews with managers of small and medium-sized manufacturing firms and industry experts indicate that low-carbon innovation in firms is closely related to stakeholders such as governments, suppliers, finance, and intermediary organizations [3], and similar results were obtained from a questionnaire survey of employees in some industrial firms [4]. Stakeholders and their actions related to carbon emissions exert pressure on some high-tech manufacturing firms, creating a driving force for low-carbon innovation [5]. Game-theoretic models demonstrate that governments’ low-carbon policies and strong environmental leadership drive firms to engage in green technological innovation [31]. Tax competition has a double-edged impact on low-carbon innovation in firms [32]. In addition to external factors, a few studies explore factors affecting low-carbon innovation from the inside. The study utilizing surveys finds that an organizational structure suitable for low-carbon innovation is a necessary condition for the success of high-rate projects [8]; literature reviews reveal that the capabilities of financiers can drive low-carbon innovation in firms [9].

Existing studies lack consideration of the impact of carbon emissions, a key internal factor, on low-carbon innovation, and may more likely follow the causal direction of the impact of low-carbon innovation on the carbon emissions of firms. Su and Moaniba (2017) [19] explored how low-carbon innovation responds to climate change in 70 countries, and the results showed that greenhouse gas emissions promote national low-carbon innovation; a spatial model study based on 30 provinces in China showed that carbon emissions accelerate ecological innovation in provinces, but the spillover effect of innovation between provinces is not obvious [33]; Wang et al. [20] further used regression methods to explore the response of provincial low-carbon innovation to climate change and found that environmental regulation plays an important intermediary role; research at the city level indicates that the increase in carbon emissions accelerate low-carbon innovation in Chinese cities, and the mechanism of influence is the improvement of public environmental awareness, which makes people inclined to purchase low-carbon and environmentally friendly consumer goods [21]. It can be seen that existing studies pay less attention to the internal factors affecting low-carbon innovation in firms. Although a few studies involve the response of low-carbon innovation to climate change, the research objects are concentrated at the regional level and have not yet paid attention to how low-carbon innovation in firms responds to climate change. At the same time, there is a lack of exploration of the internal pathways through which carbon emissions affect low-carbon innovation in firms, which is the issue that this study focuses on.

## Theoretical background and hypothesis development

Stakeholder theory holds that corporate managers need to consider the needs of all stakeholders, not just the interests of certain entities. Since stakeholders also include the natural environment and future generations who are directly or indirectly affected by the activities of firms, it is necessary to integrate the environmental social responsibility into development strategies of firms. That is, firms should not cannot just seek to maximize economic profits, otherwise they may suffer harmful consequences for their survival and development [34]. The social environment provides firms with specialized investments in production activities and undertakes certain risks, requiring high-quality responses from firms, such as protecting the environment, safeguarding the rights of employees and consumers, etc. In addition to the environment, which is directly affected by the environmental responsibility actions of firms, other stakeholders also have environmental interests and demands, exerting pressure related to carbon emissions actions on firms. Stakeholder pressure can change the decision-making of firms, such as promoting firms to pass ISO14001 certification [35], making firms aware of carbon risks and developing low-carbon innovation [36–38]. From an external perspective, stakeholders with carbon emissions-related demands such as governments, investors, and consumers will provide firms with the impetus to reduce emissions. Firms with high carbon emissions may face higher carbon pricing risks and strict supervision and regulation, compelling investors to seek higher returns as compensation, thereby escalating the capital costs in debt and equity markets [26, 39]; high carbon risk is associated with high credit risk and low investment efficiency [17, 29]; the improvement of consumers’ environmental awareness lead to a preference for low-carbon consumption, and the products and services of high-carbon-emitting firms may not meet consumer needs [21]. From an internal perspective, firms with high carbon emissions, due to their over-reliance on fossil fuels, are more vulnerable to the threat of low-cost renewable energy technology risks [26], and high technology costs may hinder firms in pursuing profit maximization, threatening the interests of internal stakeholders such as shareholders. Therefore, firms are likely to reduce costs through low-carbon innovation and gain the initiative in the market.

In summary, a high-carbon-emitting firm may face pressure from external stakeholders with carbon-related demands and internal stakeholders’ demands for transformation to reduce the risks associated with renewable energy technologies, which encourages the firm to innovate in a low-carbon direction to improve its products and services.

Based on the above analysis, this study’s first hypothesis is:

H1. Carbon emissions can drive low-carbon innovation in firms.

When firms have high carbon emissions, the uncertainty in their production and operation activities caused by the combustion of fossil fuels, namely carbon risk, is relatively high [36, 40]. That is, while firms utilize resources to generate income and value, they also bear the risk of the impact of carbon emissions on stakeholders. Among all stakeholders, governments play a significant role in exerting pressure on firms related to carbon emissions. In response to climate change, the Chinese government has established a series of robust market regulations [41, 42]. Since 2006, the Chinese government has signed energy-saving and emission reduction target responsibility letters with all provinces. An energy-saving target responsibility and evaluation assessment system has been established [43]. Provincial government work reports are increasingly emphasizing the improvement of ecological and environmental targets. Local governments will pass on emission reduction targets and pressures to firms. If firms fail to meet the low-carbon emission reduction goals, they may face substantial fines or even shutdowns. Consequently, firms with high carbon emissions may confront higher costs for not reducing emissions and pressures to do so, prompting their managers to enhance their awareness of carbon emission reduction and subsequently make decisions that are conducive to lowering carbon emissions.

After perceiving the pressure to reduce emissions, firms have two pathways to lower their carbon emissions. One is by making green investment to purchase emission reduction equipment, which can quickly achieve short-term emission reduction goals. However, because firms do not possess core technology, the associated costs are relatively high, and this approach does not achieve the long-term objective of reducing costs through emission reduction. Therefore, firms might opt for another method, which is to increase R&D investment and engage in independent innovation to achieve emission reduction. Although innovation requires substantial initial investment, it can yield long-term benefits for firms. When facing carbon risk, firms will manage to reduce costs [44]. After weighing the costs and benefits, managers are more likely to enhance their recognition of the importance of innovation [45] and increase R&D investment. Survey results from Spanish industrial firms show that when facing environmental demands from different stakeholders, firms do not respond differentially but in a similar manner, that is, by increasing R&D investment to meet the needs of stakeholders [46, 47].

Further, the resource-based theory holds that a firm’s sustained competitive advantage comes from the amount of resources it possesses [38]. Once a substantial amount of R&D investment is secured, a firm’s low-carbon innovation has a rich material basis, which is conducive to the output of low-carbon innovation. On the other hand, when a firm has a certain scale, the increased R&D investment can increase the stock of green innovation knowledge. Technicians can easily exchange innovation experiences, and the firm benefits from the "knowledge spillover" effect, leading to an improvement of green innovation efficiency.

Therefore, when faced with pressure from both internal and external stakeholders with carbon-related demands, the manager of a high-carbon-emitting firm will promote the firm to actively engage in low-carbon innovation by increasing R&D investment to achieve more low-carbon innovation outcomes.

Based on the above analysis, this study’s second hypothesis is:

H2. Carbon emissions reduction pressure is a mediator between carbon emissions and low-carbon innovation in firms.
H3. R&D investment is a mediator between carbon emissions and low-carbon innovation in firms.

## Research design

### Sample selection and data sources

Due to the disruption to corporate survival caused by the 2008–2009 global financial crisis, and the relatively complete data on low-carbon patents of listed firms before 2019, this study takes the A-share listed firms in China from 2010 to 2019 as the initial sample. The carbon emissions data of listed firms is sourced from the manual collation of annual reports, social responsibility reports, and environmental reports, etc. The data on low-carbon innovation comes from the State Intellectual Property Office (SIPO) patent publication and announcement website. Financial data is obtained from the CSMAR database. The government work reports of the provinces where listed firms are located are collected manually. This study has screened the sample as follows: (1) excluding financial listed firms; (2) excluding ST firms; (3) excluding observations with missing important research variables, and finally obtained 17,967 sample data. To prevent the impact of extreme values on the results, this study has performed a 1% winsor treatment on both sides for all continuous variables.

### Model design

#### Carbon emissions and low-carbon innovation

To test H1, this study constructs the following benchmark model:

$$LCP_{it}=\alpha_{0}+\alpha_{1}CE_{it}+\beta X_{it}+Firm_{i}+Year_{t}+\varepsilon_{it}$$ (1)

where i represents the observation listed firm, and t represents the observation year. LCP_it_ is the low-carbon innovation of listed firm i in year t, and CE is the carbon emissions of listed firm i in year t. X_it_ are the control variables. Firm_i_ is the firm-fixed effect, Year_t_ is the year-fixed effect, and ε_it_ is the error term.

#### Mediation test

To test H2 and H3, following Chen et al. (2020) [48], the following models are established for mechanism testing.


$$Pressure_{it}=\theta_{0}+\theta_{1}CE_{it}+\lambda X_{it}+Firm_{i}+Year_{t}+\varepsilon_{it}$$ (2)

$$RDI_{it}=\beta_{0}+\beta_{1}CE_{it}+\gamma X_{it}+Firm_{i}+Year_{t}+\varepsilon_{it}$$ (3)

Pressure_it_ is the mediating variable, namely, the carbon emissions reduction pressure of listed firm i in year t. Pressure1_it_ and Pressure2_it_ are specific terms that are defined in the variables section.

RDI_it_ is the mediating variable, namely, the R&D investment of listed firm i in year t.

### Variables

#### Explained variable

The dependent variable is the low-carbon innovation of listed firms (LCP). Referring to previous studies [49], this study uses the IPC codes of patent information compiled by the State Intellectual Property Office (SIPO), and distinguishes the low-carbon patent codes related to climate change mitigation from the “International Patent Classification Green List” designated by the World Intellectual Property Organization (WIPO), and matches the two to determine the number of low-carbon patents of listed firms.

#### Key explanatory variable

The main explanatory variable of this study is the carbon emissions (CE) of listed firms. There are nine categories of carbon emissions (CE) from listed firms, including emissions from the combustion of fossil fuels, emissions from the combustion of biomass fuels, fugitive emissions from raw material extraction, fugitive emissions from the oil and natural gas system, indirect carbon emissions from the import and export of electricity, emissions from the production process, emissions from the incineration of solid waste, emissions caused by wastewater treatment, and emissions resulting from the conversion of forests to industrial land due to changes in land use.

#### Control variables

According to previous studies [50–53], a series of variables that may affect low-carbon innovation are controlled. Existing research indicates that firm size affects the number of patents; therefore, the control variables in this study include firm size (Size) and the number of employees (Employees). Low-carbon innovation requires substantial long-term capital investment. A firm’s income determines the adequacy of its cash flow; hence, this study controls growth potential (Growth), business revenue (Revenue), and asset turnover rate (Turnover). Low-carbon innovation requires long-term capital investment, and the abundance of corporate capital has a significant impact on innovation. Under the condition of limited external financing, firms can leverage their comparative advantage of capital intensity for innovation activities. Therefore, this study controls the capital intensity (Capital). The characteristics of managers are linked to innovation [54, 55], thus this study also controls the proportion of shares held by managers (Mshare).

#### Mediating variables

Previous literature mostly uses pollutants, pollution fees, or costs to measure the environmental governance pressure from the government [20, 56]. However, the environmental governance pressure imposed by the government on firms is broad, encompassing not only the adjustment of pollution tax rates but also the establishment of environmental regulations and the stipulation of punitive measures, or direct administrative actions. Therefore, the aforementioned indicators may not fully reflect the carbon emissions reduction pressure on firms. Consequently, this study selects the frequency of environmental protection-related vocabulary (Pressure1) and its proportion (Pressure2) in the government work reports of the provinces where firms are located to measure the carbon emissions reduction pressure on firms. The more vocabulary used and the higher the proportion, the more the government emphasizes emission reduction, and the greater the carbon emissions reduction pressure the firms face.

This study selects the R&D investment (RDI) of listed firms as the mediating variable, with data sourced from the firms’ annual R&D expenditures. The specific variable definitions are presented in Table 1.

**Table 1:** Variable definition.

Variable	Description	Data source
**LCP**	The number of low-carbon patents	SIPO
**CE**	Carbon emissions	Hand-collected
**Growth**	Annual Revenue Growth Rate	CSMAR
**Revenue**	The natural logarithm of business revenue	
**Turnover**	Revenue to average total assets ratio	
**Size**	The natural logarithm of total assets	
**Employees**	The natural logarithm of total employees	
**Capital**	The natural logarithm of fixed assets to total employees ratio	
**Mshare**	The stock holding share of managers	
**Pressure1**	The word count related to environmental protection in the work reports of provincial governments	Hand-collected
**Pressure2**	The word frequency related to environmental protection in the work reports of provincial governments	
**RDI**	R&D expenditure	CSMAR

#### Descriptive statistics of variables

Table 2 presents the descriptive statistical results of the main variables. The average value of low-carbon innovation (LCP) is 0.7473, with a minimum value of 0.0000 and a maximum value of 735.0000, indicating a significant disparity in the levels of low-carbon innovation among the sample firms. There is also a considerable variation in carbon emissions (CE) between the sample firms.

**Table 2:** Descriptive statistics.

Variable	N	Mean	Sd	Min	Med	Max
**LCP(piece)**	17967	0.7473	10.8159	0.0000	0.0000	735.0000
**CE(one hundred thousand ton)**	17967	0.4935	3.4670	0.0000	0.0823	129.6258
**Growth**	17967	0.1849	0.4115	-0.5019	0.1136	2.6731
**Revenue**	17967	21.5242	1.4123	18.5534	21.3707	25.5190
**Turnover**	17967	2.4497	1.9169	0.4556	1.8935	12.3470
**Size**	17967	22.2109	1.3074	19.9027	22.0119	26.2207
**Employees**	17967	7.7441	1.2462	4.6250	7.6760	11.2260
**Capital**	17967	13.7900	0.9957	11.7282	13.6788	17.1757
**Mshare**	17967	0.1304	0.1994	0.0000	0.0028	0.6948
**Pressure1**	17967	8.9458	4.1146	2.0000	8.0000	21.0000
**Pressure2(%)**	17967	1.3573	0.5648	0.2951	1.3158	3.0647
**RDI(one hundred million yuan)**	17967	0.1378	0.3514	0.0000	0.0386	2.6163

## Empirical results

### Base regression results

Table 3 reports the regression results for model (1). The results in column (1) indicate that, without controlling for variables the regression coefficient of CE is significantly positive (p<0.01); column (2) shows that with the addition of control variables, the regression coefficient of CE remains significantly positive (p<0.01), indicating that carbon emissions promote low-carbon innovation in firms. The regression coefficient of CE in column (2) represents that the carbon emissions of listed firms increase by one standard deviation (3.4670) and the number of low-carbon patents increases by 0.0581 standard deviations (3.4670 × 0.1812 / 10.8159).

**Table 3:** Carbon emissions and low-carbon innovation.

Variable	LCP	
	(1)	(2)
**CE**	0.1754***	0.1812***
	(4.4920)	(4.6100)
**Growth**		-0.2179*
		(-1.9018)
**Revenue**		-0.3705
		(-1.3866)
**Turnover**		-0.1030
		(-1.4244)
**Size**		0.2228
		(0.7532)
**Employees**		0.0761
		(0.3775)
**Capital**		0.0759
		(0.4519)
**Mshare**		0.0724
		(0.1139)
**Constant**	-0.0146	1.6621
	(-0.0866)	(0.6069)
**Firm/Year**	Yes	Yes
**N**	17967	17967
**Within-R** ^**2**^	0.0056	0.0060

Note: T-statistics are reported in parentheses.

***, **, and * indicate regression coefficients significant 1%, 5%, and 10%, respectively.

### Mechanism test

Column (1) and column (2) of Table 4 report the regression results for model (2). The regression coefficient of CE is significantly positive (p<0.1), indicating that an increase in carbon emissions of firms will lead to an increase in carbon emissions reduction pressure. Column (3) of Table 4 reports the regression results for model (3). The regression coefficient of CE is significantly positive (p<0.01), indicating that an increase in carbon emissions of firms will lead to an increase in R&D investment. Thus, H2 and H3 are confirmed.

**Table 4:** The mediation effects test.

Variable	Pressure1	Pressure2	RDI
	(1)	(2)	(3)
**CE**	0.0407*	0.0057*	0.0226***
	(1.7660)	(1.7006)	(19.2572)
**Growth**	-0.1025	-0.0157	-0.0186***
	(-1.5284)	(-1.6078)	(-5.4493)
**Revenue**	0.2787*	0.0335	0.0158**
	(1.7814)	(1.4736)	(1.9822)
**Turnover**	-0.0425	-0.0073	-0.0054**
	(-1.0039)	(-1.1829)	(-2.5127)
**Size**	-0.3331*	-0.0651***	0.0566***
	(-1.9233)	(-2.5903)	(6.4185)
**Employees**	0.3405***	0.0655***	0.0279***
	(2.8855)	(3.8238)	(4.6335)
**Capital**	0.1263	0.0268*	-0.0012
	(1.2850)	(1.8813)	(-0.2430)
**Mshare**	-0.4704	-0.0547	0.0618***
	(-1.2635)	(-1.0127)	(3.2576)
**Constant**	9.1413***	1.6183***	-1.7256***
	(5.7012)	(6.9552)	(-21.1262)
**Firm/Year**	Yes	Yes	Yes
**N**	17967	17967	17967
**Within-R** ^**2**^	0.2745	0.2158	0.1891

Note: T-statistics are reported in parentheses.

***, **, and * indicate regression coefficients significant 1%, 5%, and 10%, respectively.

### Robustness test

#### Endogeneity mitigation

As previously mentioned, unlike the existing literature that examines the causal effect of low-carbon innovation on carbon emissions, the logic of this study may have an endogeneity problem of reverse causality. Therefore, the instrumental variable method is adopted to alleviate this endogeneity issue. This study uses the ranking of electricity consumption per unit GDP of the listed firms’ locations (PCP) as the instrumental variable. The smaller the electricity consumption per unit GDP is, the larger the PCP becomes. This study posits that PCP in a region is positively correlated with the carbon emissions of firms in that area, but unrelated to the low-carbon innovation in those firms. If a region has a low electricity consumption per unit GDP, it indicates that the area is less dependent on electricity, and its firms are more likely to turn to other types of mineral fuels, including coal, oil, and natural gas, to maintain production [57], resulting in low energy consumption rates and higher carbon emissions.

The instrumental variable regression results are shown in Table 5. Column (1) indicates that the coefficient of PCP in the first-stage regression is significantly positive as expected (p<0.01). Column (2) shows that the coefficient of CE in the second-stage regression is also significantly positive (p<0.05). The Kleibergen-Paap rk LM statistic is significantly positive (p<0.01), and the Kleibergen-Paap rk Wald F statistic is greater than 10, indicating the selected instrumental variable is appropriate, and the conclusion of the base regression is robust.

**Table 5:** IV regression for the carbon emissions on low-carbon innovation.

Variable	CE	LCP
	(1)	(2)
**CE**		3.2777**
		(2.3177)
**PCP**	0.0003***	
	(5.0378)	
**Growth**	0.0484**	-0.3642***
	(2.0607)	(-3.3290)
**Revenue**	0.2953***	-1.2761***
	(5.3992)	(-2.6663)
**Turnover**	0.0393***	-0.2240***
	(2.6555)	(-3.1546)
**Size**	-0.3557***	1.3135***
	(-5.8766)	(3.3799)
**Employees**	0.3137***	-0.9082***
	(7.6121)	(-2.6390)
**Capital**	0.2336***	-0.6348***
	(6.7990)	(-2.6289)
**Mshare**	0.3137**	-0.8646**
	(2.4087)	(-2.2378)
**Constant**	-4.0303***	
	(-7.1847)	
**Firm/Year**	Yes	Yes
**Kleibergen-Paap rk LM statistic**	12.252***	
**Kleibergen-Paaprk Wald F statistic**	12.276	
**N**	17967	17967
**Within-R** ^**2**^	0.0295	

Note: T-statistics are reported in parentheses.

***, **, and * indicate regression coefficients significant 1%, 5%, and 10%, respectively.

#### Alternative models

Since not all listed firms obtain low-carbon patents, this study sets up a variable SLCP, which is assigned a value of 1 if a firm has one or more low-carbon patents, and 0 otherwise. The regression results are shown in column (1) of Table 6, where the coefficient of the explanatory variable is significantly positive (p < 0.01), corroborating the conclusion of the base regression.

**Table 6:** Other robustness tests.

Variable	SLCP	LCP	FLCP	LCP
	(1)	(2)	(3)	(4)
**CE**	0.0156***		0.8750***	0.1812***
	(3.4684)		(20.5269)	(4.6083)
**LCE**		0.8774***		
		(20.5652)		
**Growth**	-0.2808***	-0.1354	-0.2234*	-0.2186*
	(-4.2637)	(-1.1008)	(-1.8573)	(-1.9013)
**Revenue**	-0.4468***	-0.5353*	-0.5591**	-0.3693
	(-3.8564)	(-1.8524)	(-1.9644)	(-1.3783)
**Turnover**	-0.3429***	-0.1151	-0.1463*	-0.1027
	(-6.2446)	(-1.4791)	(-1.8900)	(-1.4186)
**Size**	0.4888***	0.5410*	0.4968	0.2256
	(4.0095)	(1.6554)	(1.5794)	(0.7566)
**Employees**	0.4792***	-0.2533	-0.0608	0.0758
	(7.2081)	(-1.1028)	(-0.2893)	(0.3758)
**Capital**	0.3437***	-0.1744	0.0084	0.0752
	(6.2981)	(-0.9213)	(0.0476)	(0.4446)
**Mshare**	0.7765***	-0.1911	-0.2020	0.0693
	(6.0364)	(-0.2735)	(-0.2919)	(0.1083)
**Age**				-0.0102
				(-0.0893)
**Lev**				-0.0211
				(-0.0446)
**Constant**	-11.7807***	4.0261	1.6536	1.6199
	(-22.8850)	(1.3244)	(0.5531)	(0.5772)
**Firm/Year**	Yes	Yes	Yes	Yes
**N**	17967	15217	15217	17967
**Pseudo R** ^**2**^ **/Within-R** ^**2**^	0.0823	0.0363	0.0363	0.0060

Note: T-statistics are reported in parentheses.

***, **, and * indicate regression coefficients significant 1%, 5%, and 10%, respectively.

To alleviate the endogeneity issue of reverse causality, this study uses the lagged one-period CE (LCE) as the explanatory variable in model (1). The results in column (2) of Table 6 show that the coefficient of LCE is significantly positive (p < 0.01). Next, the one-period-ahead LCP (FLCP) is used as the dependent variable in model (1). The results are shown in Table 6, column (3), where the coefficient of CE is significantly positive (p < 0.01), indicating that the H1 is robust.

#### Adjustment of control variables

This study follows Chen and Yang (2019) [52] to include additional control variables of the debt level and age of a firm. The total liabilities to total assets ratio (Lev) represents the debt level of a firm and the time a firm has been in existence since its establishment (Age) represents the age of a firm. As shown in Column (4) of Table 6, the coefficient of CE is significantly positive (p < 0.01). The baseline finding is robust.

## Further discussion

### Considering the equity structure

Scholars of the law and finance school [58] believe that due to a large shareholding ratio, the controlling shareholders have the incentive to supervise the managers’ "supportive behavior" for the development of the firm, as well as the "tunneling effect" that infringes upon the interests of small and medium shareholders. Appropriately increasing the shareholding ratio of the major shareholders can make it more convenient for the major shareholders to supervise the managers for corporate governance [59]. This study speculates that the promoting effect of carbon emissions on low-carbon innovation in firms may be more significant when the concentration of equity is lower. A lower proportion of shares held by major shareholders is conducive to considering more shareholders’ opinions when making innovation decisions. Decision-making by shareholders with different experiences and professional backgrounds collectively can reduce the probability of failure in innovation decisions. This study divides the sample into a low equity concentration group (Toph = 0) and a high equity concentration group (Toph = 1) based on the median of the largest shareholder’s shareholding ratio. Columns (1) and (2) of Table 7 indicate that in the low equity concentration group (Toph = 0), the regression coefficient of CE remains significantly positive (p<0.01). In the high equity concentration group (Toph = 1), the regression coefficient of CE is not significant. The coefficients passed the inter-group difference test, indicating that when the equity concentration is lower, the promoting effect of carbon emissions on low-carbon innovation is more pronounced.

**Table 7:** The effect of the equity structure and industry nature.

Variable	LCP			
	Toph = 0	Toph = 1	High-tech = 0	High-tech = 1
	(1)	(2)	(3)	(4)
**CE**	1.6223***	0.0703	0.0305	1.1590***
	(20.7601)	(1.2247)	(0.4938)	(19.1533)
**Growth**	-0.1388	-0.2728	-0.2790	-0.2510***
	(-1.6346)	(-1.1287)	(-1.0907)	(-2.8580)
**Revenue**	-1.0864***	0.4746	0.1928	-1.0068***
	(-5.7190)	(0.7437)	(0.3176)	(-4.7232)
**Turnover**	-0.2192***	0.0980	0.0040	-0.2781***
	(-4.5633)	(0.5204)	(0.0274)	(-4.1050)
**Size**	1.2392***	-1.4428**	-0.9746	1.2012***
	(5.8726)	(-2.0257)	(-1.4403)	(5.0867)
**Employees**	-0.2597*	0.4987	0.3587	-0.2040
	(-1.8027)	(1.0847)	(0.7630)	(-1.3692)
**Capital**	-0.1450	0.3285	0.3534	-0.1224
	(-1.1749)	(0.8705)	(0.9302)	(-0.9547)
**Mshare**	-0.3854	0.1707	0.2421	-0.1222
	(-0.8015)	(0.1075)	(0.1450)	(-0.2783)
**Constant**	0.4258	12.9384*	9.8419	-0.9480
	(0.1970)	(1.8489)	(1.4319)	(-0.4485)
**Firm/Year**	Yes	Yes	Yes	Yes
**N**	9674	8293	6749	11218
**Within-R** ^**2**^	0.0654	0.0052	0.0042	0.0548
**Bdiff**	p value = 0.0000		p value = 0.0000	

Note: T-statistics are reported in parentheses.

***, **, and * indicate regression coefficients significant 1%, 5%, and 10%, respectively.

### Considering the industry nature

Firms in industries with different technological attributes exhibit significant differences in terms of innovation talent and resources. High-tech firms’ establishment and development are closely linked to technological innovation, requiring substantial capital reserves and sensitivity to the forefront of technology [60]. Their innovation strategy is more mature than that of general firms. At the same time, managers in high-tech firms have extensive experience in utilizing innovation resources and coordinating industry-university-research cooperation. When carbon emissions are high, on the one hand, high-tech firms can use their comprehensive experience in technology development to guide the generation and transformation of low-carbon innovation outcomes; on the other hand, managers of high-tech firms can use their industry experience to allocate innovation resources more efficiently when facing pressure from internal and external stakeholders. Therefore, this study speculates that the promoting effect of carbon emissions on low-carbon innovation in firms is more pronounced in high-tech firms. This study divides the sample into a non-high-tech group (High-tech = 0) and a high-tech group (High-tech = 1). Columns (3) and (4) of Table 7 show that the coefficient of CE in the non-high-tech group (High-tech = 0) is not significant, while the coefficient for the high-tech group (High-tech = 1) is significantly positive (p<0.01), confirming the speculation of this study that high-tech firms benefit more significantly from the promoting effect of carbon emissions on low-carbon innovation.

### Considering different types of carbon emissions

The role of carbon emissions in low-carbon innovation in firms is analyzed above. So, do different types of carbon emissions have the same promotional effect on low-carbon innovation? Currently, international typical carbon markets like the EU-ETS only include direct carbon emissions from the production process, known as Type 1, in the carbon emissions calculations. In contrast, many provinces and cities in China include both Type 2, which refers to indirect carbon emissions from purchased electricity, heat, and steam, and Type 1, which refers to direct carbon emissions, in their carbon emissions calculations. Previously in China, electricity prices were largely set by the government, preventing the cost of carbon emissions from being smoothly passed from the generation end to the consumer end. The original intention of the policy of including Type 2 carbon emissions in the carbon emissions is to encourage energy conservation among users. Currently, the marketization of electricity sales in China has been fully opened up, and the price signal transmission from power generation to electricity consumption has been realized. The goal of energy conservation can be achieved through market mechanisms. Incorporating Type 2 carbon emissions into carbon emissions calculations could result in "one emission, two payments" from power plants to corporate users, increasing the implicit carbon emissions costs for these firms. This may potentially increase the energy burden on firms and diminish their incentive to invest in R&D. In light of this, this study focuses on the distinct role of Type 2 carbon emissions on low-carbon innovation in firms compared to other types of carbon emissions. The paper categorizes carbon emissions data into Type 2 carbon emissions (CEID) and other types of carbon emissions (CEOT), and uses them as explanatory variables in model (1). As shown in Table 8, the effect of CEID on low-carbon innovation in firms is significantly negative (p<0.01), while the effect of CEOT on low-carbon innovation in firms is significantly positive (p<0.01), indicating that CEID inhibits low-carbon innovation. The indirect carbon emissions costs associated with purchased electricity, heat, and steam are relatively high, which restricts the ability and motivation of firms to invest in R&D for innovation.

**Table 8:** Different types of carbon emissions and low-carbon innovation.

Variable	LCP	
	(1)	(2)
**CEID**	-2.0020***	
	(-8.3790)	
**CEOT**		0.3110***
		(6.8857)
**Growth**	-0.1958*	-0.2219*
	(-1.7113)	(-1.9386)
**Revenue**	-0.2243	-0.3939
	(-0.8408)	(-1.4758)
**Turnover**	-0.0834	-0.1061
	(-1.1559)	(-1.4690)
**Size**	0.0652	0.2539
	(0.2208)	(0.8592)
**Employees**	0.2171	0.0478
	(1.0800)	(0.2374)
**Capital**	0.1796	0.0558
	(1.0718)	(0.3323)
**Mshare**	0.2041	0.0451
	(0.3216)	(0.0710)
**Constant**	-0.3869	1.9559
	(-0.1415)	(0.7150)
**Firm/Year**	Yes	Yes
**N**	17967	17967
**Within-R** ^**2**^	0.0092	0.0077

Note: T-statistics are reported in parentheses.

***, **, and * indicate regression coefficients significant 1%, 5%, and 10%, respectively.

## Conclusions and implications

In contrast to previous studies that analyze the effects of external regulation, general innovation, or green innovation on regional environmental efficiency [61, 62], this study takes an opposite approach, focusing on the response of low-carbon innovation in firms to environmental factors. Although the influencing factors of low-carbon innovation in firms are studied to some extent, they mainly concentrate on external factors such as government, institutions, carbon markets, and tax competition [3, 4, 6, 7], with a lack of research on internal factors. The research methods primarily involve conducting surveys on specific industries or small and medium-sized firms [3–5, 8], constructing game-theoretic models [31, 32], or analyzing literature [9], with a deficiency in systematic analysis using large-sample data from listed firms. This study fills this gap. Moreover, existing research is insufficient in analyzing the mechanisms affecting low-carbon innovation and has not examined the economic consequences of different types of carbon emissions. This paper is the first to study the effect of carbon emissions on low-carbon innovation in firms. It examines the logic affecting low-carbon innovation in firms from the perspective of emission reduction motives and methods, and uses data from A-share listed firms from 2010 to 2019 for verification. The research findings indicate that carbon emissions promote low-carbon innovation in firms. Mechanism analysis shows that carbon emissions reduction pressure and R&D investment are the intermediary factors through which carbon emissions influence low-carbon innovation in firms, with high-carbon-emitting firms facing pressure and increasing their R&D investment to actively engage in low-carbon innovation. Further analysis indicates that the promotional effect of carbon emissions on low-carbon innovation is more pronounced in firms with low equity concentration and in high-tech firms. Indirect carbon emissions from purchased electricity, heat, and steam do not promote low-carbon innovation, while other types of carbon emissions do. The implications of these findings are discussed below.

First, it is essential to correctly understand carbon emissions and seize the golden development period for low-carbon innovation. The research conclusions of this study indicate that firms can make endogenous responses to climate change. Carbon emissions pose a threat to global temperature rise, but they also contain the impetus for the development of low-carbon innovation. Before achieving a carbon peak, there may be a golden development period for low-carbon innovation. Governments around the world should continue to implement extensive reforms and policy stimulations, increase support for low-carbon innovation and the transformation of achievements, quickly accumulate technological advantages, and shorten the time to reach carbon neutrality. At the same time, a sound system for assessing the outcomes of low-carbon innovation and emission reduction should be established to ensure that the quantitative changes of low-carbon innovation drive qualitative achievements, truly contributing to the global climate agenda.

Second, the emission reduction regulation should be strengthened, and the climate finance system should be perfected to add impetus to low-carbon innovation through climate investment and financing. The mechanism analysis of this study indicates that the carbon emissions reduction pressure perceived by firms from governments is a mediating factor of the impact of carbon emissions on low-carbon innovation in firms. This suggests that governments should continue to focus on carbon emission reduction efforts, enforce laws strictly, and consolidate the regulatory mechanism for the quality of carbon data. On the other hand, there is a mutually beneficial prospect between green innovation and sustainable economic growth [63]. The mechanism analysis of this study indicates that carbon emissions promote low-carbon innovation in firms through increasing R&D investment. This means that efforts should be made to vigorously develop the sources of funding for low-carbon innovation. On the one hand, financial institutions should be encouraged to provide green climate funds for low-carbon innovation, improve the management level and efficiency of fund use, update green finance models in line with the times, and improve performance assessments of fund use. On the other hand, local governments should be encouraged to consider low-carbon projects as a priority in budgetary funding arrangements, assisting in the development of a low-carbon economy and sustainable development.

Third, the carbon emissions calculation system should be optimized to alleviate the burden on firms for using clean energy. Further analysis in this study indicates that indirect carbon emissions from purchased electricity, heat, and steam do not promote low-carbon innovation in firms. Including indirect carbon emissions in the carbon calculations scope reduces the relative environmental cost of fossil fuel use and increases the burden on firms for using clean energy, which is also inconsistent with international practices. Indirect carbon emissions should be excluded from the carbon emissions calculation scope to facilitate low-carbon innovation.

## Supporting information

S1 Data (XLS)
